# Intimate partner violence during the perinatal period by disability status: Findings from a United States population-based analysis

**DOI:** 10.1111/jan.15340

**Published:** 2022-06-30

**Authors:** Jeanne L. Alhusen, Genevieve Lyons, Kathryn Laughon, Rosemary B. Hughes

**Affiliations:** 1 University of Virginia School of Nursing, Charlottesville, Virginia, USA; 2 University of Virginia Public Health Sciences, Charlottesville, Virginia, USA; 3 University of Montana Rural Institute for Inclusive Communities, Missoula, Montana, USA

**Keywords:** disability, intimate partner violence, nurses, pregnancy

## Abstract

**Aims:**

The aim of the current study was to compare the prevalence of intimate partner violence (IPV) during the perinatal period among respondents with self-reported disability compared with those without a disability.

**Design:**

We conducted a secondary analysis of nationally representative data from the Pregnancy Risk Assessment Monitoring System data from 24 participating United States between 2018 and 2020.

**Methods:**

A cross-sectional sample of 43,837 respondents provided data on disability, including difficulty in vision, hearing, ambulation, cognition, communication and self-care. The exposure was perinatal IPV, defined as experiencing abuse by a current or ex-partner in the year before or during pregnancy. Regression models were used to calculated odds of IPV by disability status while accounting for relevant sociodemographic characteristics.

**Results:**

Respondents who self-reported disabilities experienced IPV at a higher rate than those without disabilities, both before and during pregnancy. In fully adjusted models, respondents with disabilities had about 2.6 times the odds of experiencing IPV before pregnancy, and about 2.5 times the odds of experiencing IPV during pregnancy, compared with those without disabilities.

**Conclusion:**

Respondents with disabilities experienced IPV at higher rates than the general population, and thus are at increased risk for adverse maternal, neonatal and infant health outcomes.

**Impact:**

Perinatal IPV is a significant issue globally, and our findings suggest perinatal IPV is particularly salient for persons with disability. Findings highlight the need to screen women with disabilities for IPV during the perinatal period as well as the importance of providing them appropriate, accessible information, resources and referrals.

## 1 | INTRODUCTION

Intimate partner violence (IPV) is a significant global public health issue, disproportionately affecting women. The prevalence of IPV varies widely across settings with global prevalence estimates demonstrating nearly 30% of women have experienced physical or sexual violence, by an intimate partner, in their lifetime (World Health Organization, 2013). Specific to the pregnancy time period, researchers have found that physical violence affects 1.2% to nearly 30% of women globally (World Health Organization, 2013). In the United States, approximately 40% of women experience some form of sexual violence in their lifetime and 20% are victims of physical violence (Smith et al., 2018). Research suggests that between 3% and 9% of women experience IPV during pregnancy (Alhusen et al., 2018; Martin et al., 2001; Saltzman et al., 2003), yet studies conducted among high-risk samples (e.g., young age, single, low socioeconomic status [SES]) have noted higher prevalence with rates up to 50% (Alhusen, Lucea, et al., 2013; Bailey & Daugherty, 2007).

Experiencing IPV in the perinatal period is associated with an increased risk for numerous adverse maternal, neonatal and infant health outcomes (Alhusen et al., 2017; Alhusen, Hayat, et al., 2013; Alhusen, Ray, et al., 2015; Saltzman et al., 2003; Samankasikorn et al., 2019; Woods et al., 2008). Risks associated with IPV in the perinatal period include inadequate prenatal care (Cha & Masho, 2014), inadequate gestational weight gain (Alhusen et al., 2017), higher rates of smoking and illicit drug use (Alhusen, Lucea, et al., 2013; Foran & O’Leary, 2008; Gilbert et al., 2012) and unintended pregnancy (Coker, 2007; Samankasikorn et al., 2019). Intimate partner violence in the perinatal period is associated with higher rates of depression (Agrawal et al., 2014; Connelly et al., 2013; Kiely et al., 2013), post-traumatic stress disorder (Huth-Bocks et al., 2013; Woods et al., 2008), suicidal ideation (Alhusen, Frohman, et al., 2015; Devries et al., 2013) and suicide and homicide (Chang et al., 2005; Martin et al., 2007; Palladino et al., 2011). In the general population, the effects of IPV extend to the consequent health of the neonate with an increased risk of low birthweight and preterm birth (Alhusen et al., 2018; Alhusen, Lucea, et al., 2013; Coker et al., 2004; Lipsky et al., 2004), both leading causes of neonatal morbidity and mortality (Bailey, 2010).

## 2 | BACKGROUND

Intimate partner violence occurring during the perinatal period may be particularly salient for the approximately 12% of women of childbearing age with disabilities living in the US, who are more likely to be single, of low SES, lack health insurance, and be unemployed, all factors associated with an increased risk of IPV (Alhusen, Ray, et al., 2015; Brown et al., 2016; Iezzoni et al., 2013; Mitra et al., 2015; Mosher et al., 2017; Okoro et al., 2018; Parish et al., 2015). Extant research demonstrates that women with disabilities are at an increased risk of violence (Alhusen et al., 2020; Breiding & Armour, 2015; Hughes et al., 2011; Mitra et al., 2012). In a nonpregnant sample, using the National Intimate Partner and Sexual Violence Survey, researchers found women with disability were significantly more likely to experience IPV compared with those without disability (Breiding & Armour, 2015). Furthermore, women with disabilities are more likely to experience multiple types of violence throughout their lives, by multiple perpetrators, and for longer durations than women without disabilities (Hughes et al., 2012; Nosek et al., 2001).

Research on the experience of IPV during the perinatal period among persons with disabilities is limited. Using data from Massachusetts Pregnancy Risk Assessment Monitoring System, Mitra et al. (2012) used a dichotomous measure of disability and found that women with disabilities were more likely to report physical abuse before pregnancy (odds ratio [OR] 4.3, 95% confidence interval [CI] 1.9–9.7), during pregnancy (OR 2.8, 95% CI 1.1–7.1) or during either time period (OR 3.2, 95% CI 1.4–7.1) than women without disabilities after controlling for relevant sociodemographics. In a qualitative study, women with disability described frequent experiences of physical, sexual and psychological IPV, ultimately resulting in unintended pregnancy (Alhusen et al., 2020). Despite our understanding of the increased risk of violence for persons with disability across the lifespan, less attention has been given to the perinatal period in particular.

## 3 | THE STUDY

### 3.1 | Aims

The purpose of this study was to examine the prevalence of IPV during the perinatal period among women with self-reported disability compared with those without a disability through the analysis of the Pregnancy Risk Assessment Monitoring System (PRAMS) data, which recently incorporated a comprehensive measure of disability as a questionnaire supplement (D’Angelo et al., 2020).

### 3.2 | Design and setting

We analysed data from Centers for Disease Control and Prevention’s (CDC’s) PRAMS, a surveillance system that collects data from the time before, during and shortly after pregnancy among persons who have had a recent live birth. PRAMS collects information on preconception health, access to health care, behaviours throughout the perinatal period, other experiences during the perinatal period (e.g. IPV, depressive symptomatology), infant health care and contraceptive use. Potential respondents are contacted between 2 and 6 months postpartum by mail. If no response by mail, they are contacted by telephone. Per CDC guidelines, data are available for states meeting a minimum response rate of greater than or equal to 65%. Survey responses are linked to birth certificate data for analyses. We used data from 2018 to 2020, the most recent data available at the time of data analysis.

### 3.3 | Participants

**FIGURE 1:**
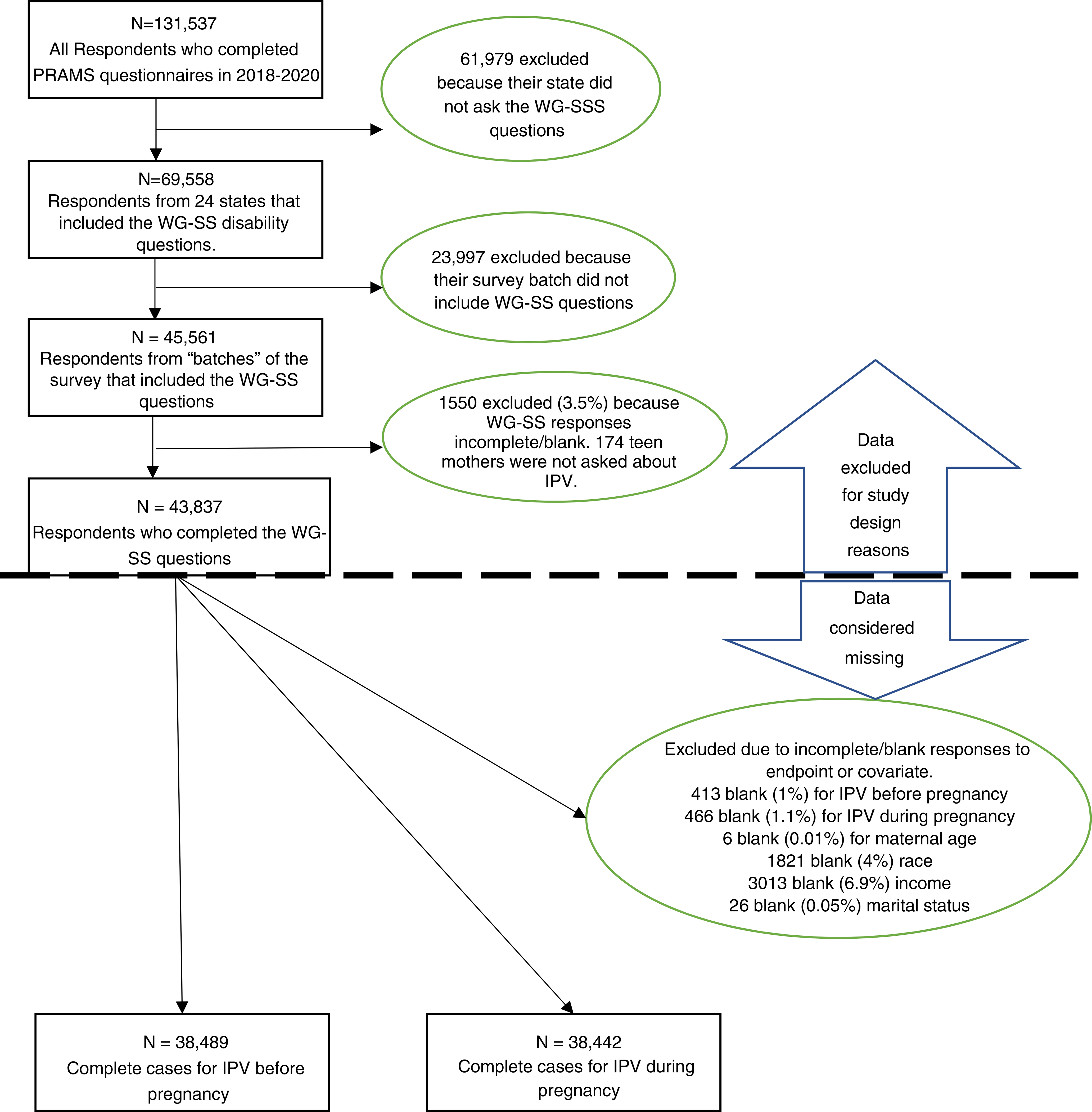
Flowchart of participant inclusion.

Each participating state selects 100–250 new persons per month, drawn from the state’s birth certificate data file, as the sampling frame. Most states oversample those persons with low birthweight infants and members of racial and ethnic minorities. Figure 1 shows a flowchart of participant inclusion. The sample for this analysis included persons who had a live birth from 2018 to 2020 (unweighted *n* = 43,811) and were asked the Washington Group Short Set of Questions on Disability (WG-SS) disability questions. The WG-SS questions were asked in 24 states, with some states discontinuing (Maine, New York, Rhode Island, West Virginia) and other states (Tennessee, New Hampshire) beginning use of WG-SS disability questions during the study period. We carefully analysed the WG-SS responses by state, by year, and by ‘batch’ for completion; our sample included only responses from batches in which the WG-SS questions were asked. Participants who were not asked the WG-SS questions were excluded from our sample, and participants who were not asked about IPV were excluded from our sample.

Overall, 24 states were included in our sample. About missing data, 406 participants (1%) had missing responses for IPV before pregnancy, and 458 participants (1.1%) had missing responses for IPV during pregnancy. Other covariates were between 0.01% and 6.9% missing; thus, we performed complete case analysis. This resulted in 35,981 complete cases to model IPV before pregnancy, and 35,935 complete cases for IPV during pregnancy.

### 3.4 | Data collection

In 2018, the disability questions were added as an optional questionnaire. The disability questionnaire supplement consists of the WG-SS that have been used in other federal and global surveys. These questions are based on the World Health Organization’s International Classification of Functioning, Disability and Health and provide standardized language as well as a framework for operationalizing disability (Svestková, 2008). Respondents are asked if they have difficulty seeing, even when wearing glasses or contact lenses; difficulty hearing, even if using a hearing aid(s); difficulty walking or climbing steps; difficulty remembering or concentrating; difficulty with self-care, such as washing or dressing; and difficulty communicating, understanding, or being understood in their usual language. Response options include no difficulty, some difficulty, a lot of difficulty and cannot do this at all. Aligned with recommendations of subject matter experts at the National Institutes of Health and CDC, and the CDC PRAMS teams, we coded a response of ‘no difficulty’ or ‘some difficulty’ as ‘no disability’ while responses of ‘a lot of difficulty’ or ‘I cannot do this at all’ were coded as ‘yes disability’. Women who answer ‘no’ to some disability questions and leave one or more of the other disability questions blank are considered missing data. The disability questions were first asked in 2018 in 22 states, but not in all ‘batches’ or months of PRAMS administration.

### 3.5 | Ethical considerations

Prior approval for this study was obtained from the institutional review board of the study team’s academic health center. As analyses included publicly available, de-identified surveillance data, this study was deemed exempt. Permission was also received from the PRAMS working group.

### 3.6 | Data analysis

The outcome variable of interest was a dichotomized measure of self-reported perinatal IPV, defined in PRAMS as being pushed, hit, slapped, kicked, choked or physically hurt by a husband or partner during the year before pregnancy or during pregnancy. Potential confounders were selected a priori given their demonstrated association with disability and perinatal IPV and included maternal age, race/ethnicity, years of education, marital status and income. Age is reported in the survey in grouped categories. Race has 11 levels in the survey and ethnicity has two (Hispanic and non-Hispanic). We collapsed race to five levels to avoid sparsity issues in less-common categories and collapsed race with ethnicity as follows: if a respondent reported Hispanic ethnicity, this superseded any other race listed. All analyses were conducted using the complex survey features of SAS v. 9.4 to account for the sampling process, design and adjusting for nonresponse and the potential for clustering around particular health care facilities, counties or time of year and provide results that are representative of the total population of mothers who gave birth to a live infant in the states/territories and time periods under study. Specifically, SAS PROC SURVEYFREQ was used for estimation of prevalence and confidence intervals the Rao-Scott chi-square test was used to test for significant difference in prevalence of IPV by group, and PROC SURVEYLOGISTIC was used to run logistic regression models and estimate ORs for IPV with adjustment for covariates. Separate models were estimated for IPV before pregnancy and IPV during pregnancy.

### 3.7 | Validity, reliability and rigour

The PRAMS is one of the largest state-based surveillance data sets of women with live births, including their experiences before, during and after pregnancy. Data obtained from PRAMS are linked to birth certificate information. Because PRAMS data are self-reported, the reliability and validity with other population-based data collection systems, such as the birth certificate, have been established in multiple studies (Ahluwalia et al., 2013; Gayle et al., 1988; Hosler et al., 2010). All questions are developed and pretested by the CDC or participating states. Standardization of data collection procedures and measures between states allows for national as well as state-by-state comparisons (Centers for Disease Control and Prevention, 2007).

## 4 | RESULTS

The sociodemographics of study respondents are shown in Table 1. Disability data were available on 43,811 respondents. With regards to disability, 6.5% of respondents reported at least one type of disability (95% CI 6.1–6.9). Respondents with at least one disability were significantly more likely to report less than a high school education with 17.3% (95% CI 14.8–19.7) of respondents with at least one type of disability reporting less than a high school education compared with 10.7% (95% CI 10.1–11.2) if respondents without a disability. Relatedly, 50.7% (95% CI 47.4–53.9) of respondents with at least one disability reported an income <100% Federal Poverty Level compared with 26.9% (95% CI 26.1–27.7) of respondents without a disability. With regards to race/ethnicity, respondents with at least one disability had a lower proportion of White and Asian races, compared with respondents without a disability. Finally, 62.2% (95% CI 61.4–63.0) of respondents without disabilities reported being married while 42.2% (95% CI 39.1–45.3) of respondents with a disability reported being married.

The prevalence of IPV by disability status is shown in Table 2. A Rao-Scott chi square test indicated a significant association between disability status and IPV before and during pregnancy (*p*-values <.001 and <.001, respectively). In the time before pregnancy, the prevalence of IPV for respondents with disability was 9.5% (95% CI 7.8–11.2), compared with those with no disability, who had a prevalence of 2.4% (95% CI 2.1–2.6). Similar significant differences in the prevalence of IPV by disability status were noted during pregnancy. Specifically, the prevalence of IPV during pregnancy was 5.8% (95% CI 4.4–7.2) for respondents with a disability compared with 1.7% (95% CI 1.4–1.9) for respondents without a disability.

Table 3 presents the results from the multivariable logistic regression models. Covariates included in the full models were age, educational attainment, race, household income and relationship status. In fully adjusted models, respondents with disability were significantly more likely to experience IPV before pregnancy (adjusted odds ratio [aOR] 2.6, 95% CI 2.1–3.4) and during pregnancy (aOR 2.5, 95% CI 1.8–3.4) compared with respondents without disability. This corresponds to more than twice the odds of IPV among respondents with disabilities, both before and during pregnancy, after adjusting for other factors. In adjusted models, other covariates significantly associated with IPV before pregnancy were educational attainment, race, household income and relationship status. During pregnancy, other covariates associated with IPV were educational attainment, household income and relationship status. Race was not associated with IPV in the presence of the other covariates.

## 5 | DISCUSSION

The results of this population-based study indicate that women with disabilities were more than twice as likely as women without disabilities to report IPV occurring before and during pregnancy even after controlling for relevant sociodemographic characteristics. Having less than a college education, identifying as white race, or having a household income below 200% of the federal poverty line were associated with a higher prevalence of perinatal IPV in our sample. Of note, education, race and ethnicity and income also reflect well-known sociodemographic disparities experienced by women with disabilities in the United States regardless of pregnancy status (Mosher et al., 2017; Okoro et al., 2018).

Our study represents the first known analysis of PRAMS data collected in multiple states in the United States to examine those associations. Our findings on perinatal IPV disparities experienced by women with disabilities are consistent with those reported by Mitra et al. (2012). Among the first to address this nascent area of research, that study was limited to PRAMS data collected in only a single state versus multiple states and did not explore potential socioeconomic risks of perinatal IPV as examined in the current study. Our findings also confirm other research showing that during the perinatal period, women with disabilities experience IPV around the time of pregnancy. These studies strongly suggest that perinatal IPV is a substantial problem for women with disabilities, requiring attention by providers with disability-related knowledge and experience.

Given the disparities shown by the present study, screening women with disabilities for IPV during the perinatal period is an important step toward preventing the occurrence of serious injury, additional disabling health conditions, or death. In 2012, the American College of Obstetricians and Gynaecologists (ACOG) released a practice opinion highlighting the importance of physicians screening all women for IPV at periodic intervals, including during obstetric care (i.e., at the first prenatal visit, at least once per trimester, and at the postpartum checkup) (‘ACOG Committee Opinion No. 518: Intimate Partner Violence’, 2012). ACOG offered sample screening questions including ‘Has your partner ever hit, choked, or physically hurt you?’ and ‘Has your partner ever threatened you or made you feel afraid?’ Of particular relevance to our study, ACOG also noted vulnerabilities for abuse among women with disabilities and suggested disability-sensitive IPV screening questions. Our results may underestimate the differences between women with and without disabilities on perinatal IPV prevalence. Intimate partners of women with disabilities can also perpetrate physical IPV in the form of physical restraint or confinement in inaccessible locations; withholding needed wheelchairs and other equipment, medications or transportation; or refusing to assist with essential daily living needs, such as eating or getting out of bed. Thus, without ever touching a woman, an abusive partner’s behaviour could result in physical harm leading to adverse maternal, neonatal and other outcomes. We recommend that future research addressing perinatal IPV and women with disabilities incorporate the examples of screening questions published by ACOG such as ‘Has your partner prevented you from using a wheelchair, cane, respirator or other assistive device?’ and ‘Has your partner refused to help you with an important personal need such as taking your medicine, getting to the bathroom, getting out of bed, bathing, getting dressed or getting food or drink or threatened not to help you with these personal needs?’ (‘ACOG Committee Opinion No. 518: Intimate Partner Violence’, 2012).

### 5.1 | Limitations

Our findings are subject to at least five limitations. First, because PRAMS relies on a retrospective self-report of perinatal IPV, the findings may be subject to recall bias, misunderstanding of questions, nonresponse and other biases. Second, the PRAMS item assessing IPV asks about ‘being hit, slapped, choked or physically hurt by a husband, or partner’, which does not adequately reflect the types of IPV that pregnant and postpartum persons may be experiencing, including psychological and sexual violence. Previous research suggests sexual and psychological violence may be particularly salient for pregnant persons with disabilities (Alhusen et al., 2020). Third, the IPV question used in this study was restricted to husband or partner, which may not fully capture the nature of violence unique to women with disabilities whose perpetrators are known to include not only intimate partners but also caregivers, health care professionals, transportation drivers or friends and family members. Fourth, because the IPV questions and/or the disability questions were only asked in certain states, and not in all batches of PRAMS, our study is subject to an administrative limitation whereby observable information is left unobserved. Nonetheless, we achieved consistent and significant results. To further advance the understanding of perinatal IPV in the context of disability, future research could seek to replicate our findings with a larger sample. Finally, our analysis was limited to respondents in the United States. IPV is a global issue and these relationships should be examined in all contexts.

Despite its limitations, our analyses yielded a prevalence of IPV that was significantly higher among mothers with disability than those without disabilities, both before and during pregnancy. Regardless of how we approach the experience of perinatal IPV, it is clear that disability introduces unique dimensions to the experience of violence against women around the time of their pregnancies. It is important that health care providers consider disability-specific forms and perpetrators of perinatal IPV when assessing women with disabilities. Our findings are also subject to the limitations of the WG-SS questions, which are limited in the types of disabilities detected.

## 6 | CONCLUSION

Results of this study have clinical implications for perinatal care providers. Our findings highlight the importance of screening all women for IPV before and during pregnancy with particular attention given to those who have less education, membership in racial minority groups and/or income falling below the federal poverty line. The disability disparities shown by our analyses substantiate the need to screen women with disabilities for IPV during the perinatal period as well as the importance of providing them appropriate, accessible information, resources and referral. Addressing the disproportionate prevalence of perinatal IPV experienced by women with disabilities can reduce risks for adverse maternal and infant outcomes and occurrence of additional disabling injuries and health conditions, as well as exert positive effects on the overall health and safety in this seriously disadvantaged, marginalized and medically underserved population of women.

## Figures and Tables

**TABLE 1:** Respondent characteristics

	No disability reported (unweighted *n* = 70,813, weighted *n* = 1,810,916)		At least one disability (unweighted *n* = 3024, weighted *n* = 112,200)	
Characteristic	Column %	95% CI	Column %	95% CI
Age* (years)				
<20	3.80	(3.47, 4.13)	5.48	(4.12, 6.83)
20–24	17.75	(17.09, 18.40)	26.45	(23.64, 29.25)
25–34	59.68	(58.86, 60.49)	52.72	(49.61, 55.84)
35+	18.78	(18.15, 19.41	15.35	(13.12, 17.59)
Education level*				
<High school	10.69	(10.14, 11.23)	17.27	(14.79, 19.74)
High school	24.93	(24.17, 25.69)	36.87	(33.75, 39.99)
Some college	25.56	(24.86, 26.26)	30.81	(28.09, 33.54)
Bachelors+	38.82	(38.04, 39.60)	15.05	(12.85, 17.26)
Race**				
Asian	4.35	(4.02, 4.67)	2.97	(2.03, 3.91)
White	58.06	(57.29, 58.84)	54.98	(51.87, 58.10)
Black	18.81	(18.18, 19.44)	21.62	(19.06, 24.20)
Other/mixed	3.57	(3.29, 3.85)	4.41	(3.34, 5.47)
Hispanic	15.20	(14.60, 15.80)	16.01	(13.59, 18.43)
Income^a^,*				
<100% Federal poverty level (FPL)	26.89	(26.12, 27.66)	50.66	(47.41, 53.91)
101%-200% FPL	22.11	(21.38, 22.83)	24.59	(21.81, 27.36)
>200% FPL	51.00	(50.16, 51.84)	24.75	(22.03, 27.48)
Relationship status*				
Married	62.20	(61.39, 63.00)	42.22	(39.12, 45.33)
Other	37.80	(37.00, 38.61)	57.78	(54.67, 60.88)

Abbreviation: CI, confidence interval.

a FPL depends on income and household size. Income was reported in ranges. Respondents’ %FPL was calculated using the midpoint of their reported income range and their reported number of dependents.

* Rao-Scott *p*-value <.001

** Rao-Scott *p*-value <.05.

**TABLE 2:** Prevalence of intimate partner violence (IPV) by disability status

**Disability status**	**IPV before pregnancy** *	**Frequency**	**Weighted frequency**	**Percent**	**95% confidence limits for percent**	
					
No disability	No IPV	39,288	1,755,009	97.61	97.36	97.86
	IPV	1161	43,000	2.39	2.14	2.64
At least one disability	No IPV	2645	112,130	90.52	88.80	92.24
	IPV	330	11,745	9.48	7.76	11.20
**Disability status**	**IPV during pregnancy** *	**Frequency**	**Weighted frequency**	**Percent**	**95% confidence limits for percent**	
					
No disability	No IPV	39,578	1,766,551	98.35	98.14	98.56
	IPV	814	29,585	1.65	1.44	1.86
At least one disability	No IPV	2773	116,790	94.18	92.78	95.59
	IPV	206	7212	5.82	4.41	7.22

* Rao-Scott chi-square *p* < .001.

**TABLE 3:** Adjusted logistic regression model for intimate partner violence (IPV) before pregnancy and during pregnancy

	IPV before pregnancy^a^			IPV during pregnancy^b^		
Characteristic	Odds ratio	95% CI	*p*-value	Odds ratio	95% CI	*p*-value
Disability status (ref = No disability)						
At least one disability	**2.64**	**(2.06, 3.39)**	**<.0001**	**2.46**	**(1.81, 3.35)**	**<.0001**
Age group (ref = age 25–34)						
<20	0.83	(0.53, 1.29)	.4089	0.77	(0.44, 1.35)	.3644
20–24	1.09	(0.86, 1.38)	.4951	1.26	(0.94, 1.70)	.1205
35+	1.01	(0.73, 1.38)	.9695	0.83	(0.56, 1.24)	.3621
Educational attainment (ref = Bachelors+)						
<High school	**2.02**	**(1.26, 3.25)**	**.0037**	**2.08**	**(1.15, 3.77)**	**.0151**
High school	**1.69**	**(1.09, 2.60)**	**.0186**	1.49	(0.87, 2.57)	.1495
Some college	**1.79**	**(1.20, 2.67)**	**.0041**	**1.68**	**(1.04, 2.711)**	**.0335**
Race (ref = White non-Hispanic)						
Asian	**0.38**	**(0.18, 0.78)**	**.0084**	0.61	(0.28, 1.31)	.2029
Black	**0.67**	**(0.52, 0.86)**	**.0017**	1.16	(0.85, 1.57)	.3484
Hispanic	**0.64**	**(0.47, 0.88)**	**.0053**	0.71	(0.49, 1.03)	.0677
Other/mixed	1.30	(0.94, 1.79)	.1141	1.18	(0.83, 1.69)	.3604
Household income (ref = >200% FPL)						
<100% FPL	**3.67**	**(2.45, 5.49)**	**<.0001**	**4.07**	**(2.54, 6.53)**	**<.0001**
101%-200% FPL	**1.90**	**(1.26, 2.87)**	**.0024**	**2.14**	**(1.34, 3.43)**	**.0016**
Relationship status (ref = Married)						
Other	**2.76**	**(2.06, 3.70)**	**<.0001**	**2.08**	**(1.47, 2.95)**	**<.0001**

Bolded values have reached statistical significance.

Abbreviations: CI, confidence interval; FPL, federal poverty level.

a *n* = 38,489.

b *n* = 38,442.

## Data Availability

The data that support the findings of this study are available via PRAMS at https://www.cdc.gov/prams/index.htm.
